# Dynamic Proteomic and Metabolomic Analysis Reveals Metabolic Reprogramming During Early Neuronal Transdifferentiation of Human Fibroblasts Driven by Forskolin

**DOI:** 10.3390/cimb48090871

**Published:** 2026-08-27

**Authors:** Xiang Yuan, Siyao Pan, Zhiqiang Wang, Dandan Zhang, Guodong Wang, Ben Huang

**Affiliations:** Guangxi Zhuang Autonomous Region Engineering Research Center for 3D Printing in Smart Biomanufacturing and Application, Guangxi Academy of Medical Sciences, Nanning 530021, China; xyuan@gxams.org.cn (X.Y.);

**Keywords:** forskolin, cell transdifferentiation, proteomics, metabolic remodeling

## Abstract

Forskolin (FSK) is a well-characterized small-molecule activator of adenylyl cyclase that drives direct neuronal transdifferentiation in human fibroblasts; however, the temporal sequence and coordinated relationships among proteomic and metabolic adaptations during the initiation phase of lineage conversion remain poorly understood. In this study, we applied data-independent acquisition (DIA)-based quantitative proteomics and untargeted metabolomics on BJ human dermal fibroblasts at three biological timepoints: pre-induction (day 0), commitment onset (day 2), and neuronal maturation (day 5). Under the established FSK-based induction protocol, BJ fibroblasts rapidly acquired neuronal-like features, with more than 90% of cells becoming TUJ1-positive by day 5. Proteomic profiling revealed a profound, dichotomous regulatory shift: time-dependent activation of core metabolic and energy pathways (glycolysis, the TCA cycle, and oxidative phosphorylation) coupled with persistent suppression of cell-cycle progression and DNA replication. Concordantly, global metabolomic profiling revealed a statistically unidirectional transition in metabolic states characterized by the progressive accumulation of phosphoenolpyruvate (PEP). Collectively, our findings identify coordinated remodeling of central carbon metabolism as a prominent early molecular feature associated with neuronal transdifferentiation under the FSK-based induction protocol. This study provides an integrated proteomic and metabolomic framework for understanding early molecular remodeling during chemically induced cell fate conversion and provides a basis for future functional studies of metabolic regulation during reprogramming.

## 1. Introduction

Neurons serve as indispensable functional units for maintaining physiological homeostasis and executing higher-order neural computations. Owing to their terminally differentiated and post-mitotic state, mature neurons exhibit virtually no capacity for self-renewal or de novo regeneration. Consequently, neuronal loss, whether triggered by acute insults or chronic neurodegenerative diseases, leads to enduring and debilitating impairments in motor coordination, cognitive function, and sensory perception [1,2,3]. To date, no clinically approved intervention has restored lost neuronal circuitry. In recent years, somatic cell reprogramming has emerged as a revolutionary strategy for neuronal regeneration, enabling direct conversion of somatic cells, such as fibroblasts, into functional neurons via transcription factor or small-molecule induction [4,5,6]. However, viral-vector-mediated reprogramming carries inherent risks of genomic integration and insertional oncogenesis [7,8,9]. Although chemically induced neuronal reprogramming (CiN) avoids these genotoxic hazards, it typically relies on empirically optimized, multi-component “cocktail” formulations [10,11]. The mechanistic basis for inter-compound synergy remains poorly understood, and cumulative cytotoxic effects pose significant safety concerns [12]. Collectively, these limitations impede the scalability, reproducibility, and clinical translation of CiN.

Harnessing a single small molecule is a compelling approach to neuronal reprogramming. Forskolin (FSK), a safe and highly permeable diterpenoid, activates adenylate cyclase (AC) to elevate intracellular cAMP levels and engage the PKA/CREB signaling axis [13,14]. We previously demonstrated that FSK alone efficiently reprograms human fibroblasts into functional induced neurons (FiNs). These FiNs are viable, electrophysiologically mature, and functionally integrate into host circuits both in vitro and in vivo. Mechanistically, this transdifferentiation is predominantly driven by the cAMP–CREB1–JNK cascade [15]. Nevertheless, the comprehensive regulatory architecture governing FiNs’ specification remains unclear. Notably, the role of cellular metabolic reprogramming in this single-molecule transition has yet to be systematically investigated.

To systematically dissect the molecular architecture underlying FSK-driven neuronal transdifferentiation, we conducted time-resolved, integrative proteomic and metabolomic profiling of human somatic cells at three critical transition points: days 0, 2, and 5. Integrated proteomic and metabolomic analyses uncovered coordinated increases in glycolytic enzymes (ALDOB and ENO2) and core components of the TCA cycle (ACO2, IDH1, OGDH, SUCLA2, and MDH2), together with corresponding changes in central carbon metabolites. Notably, levels of phosphoenolpyruvate (PEP), a key metabolite at the glycolytic–mitochondrial interface, surged by day 2 and remained elevated, serving as an early and sustained metabolic feature associated with the cell state transition. These metabolic changes emerged early and occurred in parallel with the progressive acquisition of neuronal markers, suggesting a close temporal association between metabolic remodeling and transdifferentiation. Our findings identify bioenergetic remodeling as a prominent molecular component of early chemical reprogramming and provide a framework for subsequent functional investigation.

## 2. Materials and Methods

### 2.1. Cells and Cell Culture

#### 2.1.1. Cell Culture

Human skin fibroblast BJ cells (ATCC, Manassas, VA, USA, catalog No. CRL-2522) were cultured in a fibroblast medium (FM) consisting of DMEM (Gibco, Grand Island, NY, USA, 11965-092) supplemented with 10% fetal bovine serum (FBS; WISENT, Montreal, QC, Canada, 086-150). For passaging, cells were rinsed three times with PBS (Beyotime, Shanghai, China, C0221A) and briefly digested with trypsin (Gibco, Grand Island, NY, USA, 25200-072) at room temperature for 1 min. An equal volume of the FM was added to neutralize trypsin activity, and dissociated cells were reseeded at a 1:3 split ratio onto new culture plates.

#### 2.1.2. Generation and Culture of Somatic Cell-Derived Induced Neurons

BJ fibroblasts were plated onto poly-D-lysine (PDL; Gibco, Grand Island, NY, USA, A38904-01)-coated culture dishes and maintained in FM until reaching 80% confluency. The original medium was then replaced with neuronal induction medium (IM).

At day 2 post-induction (D2), IM was substituted with a neuronal maturation medium (MM), with medium refreshment performed every two days thereafter. On day 5 (D5), 10 μM forskolin was withdrawn from the culture system, and cells were cultured in the neuronal maintenance medium (NM), which was renewed every other day.

#### 2.1.3. Medium Formulations

Neuronal Induction Medium (IM): DMEM/F12 (Life Technologies, Grand Island, NY, USA, 11330-032) and Neurobasal (Life Technologies, Grand Island, NY, USA, 21103-049) mixed 1:1, supplemented with 0.5% N2 (Invitrogen, Grand Island, NY, USA, 17502048), 1% B27 (Invitrogen, Grand Island, NY, USA, 17504044), 20% KnockOut™ Serum Replacement (Gibco, Grand Island, NY, USA, 10828-028), 10 μM FSK, 100 μM cAMP, 20 ng/mL bFGF, and penicillin/streptomycin.

Neuronal Maturation Medium (MM): DMEM/F12:Neurobasal (1:1), 0.5% N2 (Invitrogen, Grand Island, NY, USA, 17502048), 1% B27 (Invitrogen, Grand Island, NY, USA, 17504044), 10 μM FSK, 100 μM cAMP, 20 ng/mL bFGF, 20 ng/mL BDNF, 20 ng/mL GDNF, 20 ng/mL NT3, and penicillin/streptomycin.

Neuronal Medium (NM): DMEM/F12:Neurobasal (1:1), 0.5% N2 (Invitrogen, Grand Island, NY, USA, 17502048), 1% B27 (Invitrogen, Grand Island, NY, USA, 17504044), 20 ng/mL BDNF, 20 ng/mL GDNF, 20 ng/mL NT3, and penicillin/streptomycin.

### 2.2. Immunofluorescence Staining

Cells were washed thrice with phosphate-buffered saline (PBS), fixed in 4% paraformaldehyde for 20 min, and blocked in PBS containing 100 mM glycine and 0.3% BSA (3 × 5 min). After an additional 1.5 h block with 1% BSA, cells were incubated with primary antibodies overnight at 4 °C, followed by secondary antibodies for 1.5 h at room temperature. Nuclei were counterstained with DAPI (Thermo Fisher Scientific, Waltham, MA, USA, Cat. #D1306) (1 μg/mL in PBS) for 10 min, and unbound dye was removed via three final PBS washes. Imaging was performed using a fluorescence microscope. Primary antibodies: anti-TUJ1 (1:500; Covance, Princeton, NJ, USA, Cat. #MMS435P) and MAP2 (1:500; CST, Danvers, MA, USA, Cat. #4542s). Secondary antibodies: Alexa Fluor 488 donkey anti-mouse (1:200; Abcam, Cambridge, UK, Cat. #ab150109) and Alexa Fluor 555 donkey anti-rabbit (1:200, Abcam, Cambridge, UK, Cat. #ab150074) antibodies.

Neuronal differentiation efficiency was determined by counting TUJ1-positive cells exhibiting a typical neuronal morphology and DAPI-stained nuclei in 5–10 randomly selected fields per replicate. The ratio of TUJ1-positive cells to total nuclei was calculated. Experiments were performed in triplicate, and mean values were reported for analysis.

### 2.3. Western Blot Analysis

Cells were lysed in ice-cold RIPA lysis buffer (Servicebio, Wuhan, China, #G2002-100ML) supplemented with protease and phosphatase inhibitors (Servicebio, Wuhan, China, #G2006-250UL, #G2007-1ML). Total protein concentration was quantified using the bicinchoninic acid (BCA) assay (Servicebio, Wuhan, China, #G2026-200T). A total of 30 µg of total protein was resolved by SDS-polyacrylamide gel electrophoresis (SDS-PAGE) under reducing conditions and electrotransferred onto polyvinylidene fluoride (PVDF) membranes. After blocking, membranes were incubated overnight at 4 °C with primary antibodies against the indicated target proteins, including TUBA1B (1:10,000, Proteintech, Rosemont, IL, USA, #11224-1-AP), ENO2 (1:1000, Servicebio, Wuhan, China, #GB150204-100), MDH2 (1:2000, Servicebio, Wuhan, China, #GB114349-100), ACO2 (1:2000, Servicebio, Wuhan, China, #GB113832-100), Notch3 (1:500, Servicebio, Wuhan, China, #GB111310-100), IDH1 (1:500, Servicebio, Wuhan, China, #GB111194-100), and Smad3 (1:2000, Servicebio, Wuhan, China, #GB150085-100). The membranes were then incubated with horseradish peroxidase (HRP)-conjugated goat anti-rabbit secondary antibody (1:50,000, Servicebio, Wuhan, China, #GB23303). Protein bands were detected using enhanced chemiluminescence (ECL) reagents (Servicebio, Wuhan, China, #G2020-1) and quantified using the ImageJ v1.54g software, with TUBA1B serving as the internal loading control.

### 2.4. Protein Analysis

#### 2.4.1. Total Protein Extraction

Frozen cell pellets were rapidly thawed on ice and lysed in denaturing lysis buffer (8 M urea, 1% SDS, Thermo Scientific, Waltham, MA, USA). Homogenization was performed using a high-throughput tissue grinder (three cycles of 180 s each, with intermittent cooling on ice), and then non-contact cryogenic sonication (30 min at 4 °C; duty cycle 5%, amplitude 30%) was performed to ensure complete disruption of nuclear envelopes and efficient solubilization of chromatin-bound and membrane-associated proteins. Lysates were centrifuged at 16,000× *g* for 30 min at 4 °C to remove insoluble cellular debris, aggregated protein complexes, and residual genomic DNA. The protein concentration in the clarified supernatant was quantified using the BCA assay according to the manufacturer’s protocol. Equal protein amounts were subjected to SDS-PAGE.

#### 2.4.2. Protein Digestion

A total of 100 µg of total protein was diluted in 100 mM triethylammonium bicarbonate (TEAB) buffer (pH 8.5). The sample lysate was reduced with 10 mM tris (2-carboxyethyl) phosphine (TCEP) at 37 °C for 60 min and then subjected to alkylation with 40 mM iodoacetamide (IAM) in the dark at room temperature for 40 min. After centrifugation (10,000× *g*, 4 °C, 20 min), the precipitated protein pellet was resuspended in 100 µL of 100 mM TEAB buffer. Trypsin (Promega, Madison, WI, USA, sequencing-grade, V5111) was added at an enzyme-to-substrate mass ratio of 1:50, and digestion was carried out overnight at 37 °C under gentle agitation.

#### 2.4.3. Peptide Desalting and Quantification

Peptides were dried under vacuum centrifugation and reconstituted in 0.1% (*v*/*v*) trifluoroacetic acid (TFA). Desalting was performed using Waters Oasis HLB µElution cartridges (10 µg capacity, Waters, Milford, MA, USA), following the manufacturer’s recommended protocol. Prior to DIA analysis, the peptide concentration was determined by UV absorbance at 280 nm using a NanoDrop One spectrophotometer (Thermo Scientific, Waltham, MA, USA).

#### 2.4.4. Data-Independent Acquisition (DIA) Mass Detection

Peptide samples were analyzed on a Vanquish Neo UHPLC system (Thermo Scientific, Waltham, MA, USA) coupled with a Compass HyStar platform (Bruker Daltonik GmbH, Bremen, Germany) at Majorbio Bio-Pharm Technology Co., Ltd. (Shanghai, China). Peptides were separated on a self-packed C18 column (100 µm × 15 cm, 1.7 µm particles) using a linear gradient of solvent A (0.1% formic acid and 2% acetonitrile in water) and solvent B (0.1% formic acid and 80% acetonitrile in water) over 8 min at a flow rate of 300 nL/min. Data-independent acquisition (DIA) mass spectra were acquired on a timsTOF HT mass spectrometer (Bruker Daltonik GmbH, Bremen, Germany) operating in DIA mode. The MS1 spectra were acquired within an *m*/*z* range of 70–1050, while MS2 spectra were collected over an *m*/*z* range of 150–2000.

#### 2.4.5. Protein Identification and Quantification

DIA raw files were processed using Spectronaut v19. The database adopted in this study was the Ensembl database (release 113, based on genome assembly GRCh38.p13, https://ftp.ensembl.org/pub/release-113/fasta/homo_sapiens/pep/Homo_sapiens.GRCh38.pep.abinitio.fa.gz, accessed on 9 April 2025). The search parameters included the following: peptide length—7–52 residues; digestion specificity—trypsin/P; up to two missed cleavages; fixed modification—carbamidomethylation of cysteine; and variable modifications—oxidation of methionine and acetylation of protein N-terminus. The false-discovery rate (FDR) was kept at ≤1% at both protein and peptide levels (q-value ≤ 0.01); the minimum peptide confidence threshold was ≥99%; and the XIC integration window was ≤75 ppm. Protein abundance was quantified using MaxLFQ normalization.

#### 2.4.6. Statistical and Functional Analysis

Differentially expressed proteins (DEPs) were identified using the R package (limma, v3.60.0). Significance thresholds were defined as FC > 1.5 or FC< 0.67 and *p*-values < 0.05. Functional annotation of all identified proteins was performed using the Gene Ontology (GO) resource (http://geneontology.org/, accessed on 20 June 2025) and the Kyoto Encyclopedia of Genes and Genomes (KEGG) pathway database (http://www.genome.jp/kegg/, accessed on 20 June 2025). DEPs were subsequently subjected to KEGG enrichment analyses.

### 2.5. Metabolomics Analysis

#### 2.5.1. Sample Preparation

Samples were homogenized in 2 mL centrifuge tubes using 6 mm stainless-steel grinding beads and 300 μL of ice-cold methanol–water (4: 1, *v*/*v*) extraction solvent containing four internal standards, including 0.02 mg/mL of L-2-chlorophenylalanine. We performed cryogenic milling at −10 °C and 50 Hz for 6 min, followed by low-temperature ultrasonication at 5 °C and 40 kHz for 30 min. The extracts were then incubated at −20 °C for 30 min to precipitate proteins and lipids and centrifuged at 13,000× *g* and 4 °C for 15 min, and the clarified supernatants were collected for analysis. A pooled quality control (QC) sample was prepared by combining equal-volume (20 μL) aliquots from the supernatant of each biological sample.

#### 2.5.2. UHPLC-MS/MS Analysis

Chromatographic separation was performed using a Thermo Scientific UHPLC-Orbitrap Exploris 240 system (Thermo Scientific, Waltham, MA, USA) coupled to an ACQUITY UPLC HSS T3 column (100 mm × 2.1 mm, 1.8 μm, Waters, Milford, MA, USA) maintained at 40 °C, with an injection volume of 3 μL. Mobile phase A consisted of 95% water and 5% acetonitrile (both containing 0.1% formic acid); mobile phase B consisted of 47.5% acetonitrile, 47.5% isopropanol, and 5% water (all containing 0.1% formic acid). Mass spectrometry was conducted utilizing an electrospray ionization (ESI) source operating in both positive and negative ion modes. Data were acquired over a mass range of *m*/*z* 70–1050 with a mass resolution of 60,000 for MS1 and 15,000 for MS2. The source parameters were set as follows: sheath gas flow rate, 60 arbitrary (arb) units; auxiliary gas flow rate, 20 arb; heater temperature, 350 °C; capillary temperature, 320 °C; spray voltage, 3400 V for positive mode and −3000 V for negative mode; S-Lens RF level, 70; and normalized collision energy (NCE), 20%, 40%, and 60%. To monitor the stability of the analytical system, one QC sample was interspersed every 5 to 15 experimental samples throughout the acquisition sequence.

#### 2.5.3. Data Processing and Metabolite Identification

The raw MS data were processed using the Progenesis QI v3.0 software for peak picking, retention time alignment, integration, and baseline correction. Metabolite identification was achieved by matching accurate monoisotopic masses (mass accuracy < 10 ppm) and diagnostic MS^2^ fragment ions against reference spectra in HMDB, METLIN, and a laboratory-curated spectral library. Unsupervised principal component analysis (PCA) and supervised orthogonal partial least-squares discriminant analysis (OPLS-DA) were performed to assess global metabolic variation and maximize class separation, respectively. Differential metabolites were identified based on a variable importance in projection (VIP) score > 1.0 from partial least-squares discriminant analysis (PLS-DA) and a *p*-value < 0.05.

#### 2.5.4. KEGG Annotation and Enrichment Analysis of Differential Metabolites

The identified metabolites were annotated using the KEGG Compound database (https://www.kegg.jp/kegg/compound/, accessed on 28 June 2025) and subsequently mapped to relevant KEGG pathways (https://www.kegg.jp/kegg/pathway.html, accessed on 28 June 2025) with the KEGG Mapper tool (https://www.kegg.jp/kegg/mapper/, accessed on 28 June 2025).

## 3. Results

### 3.1. Morphological Characterization

**Figure 1:**
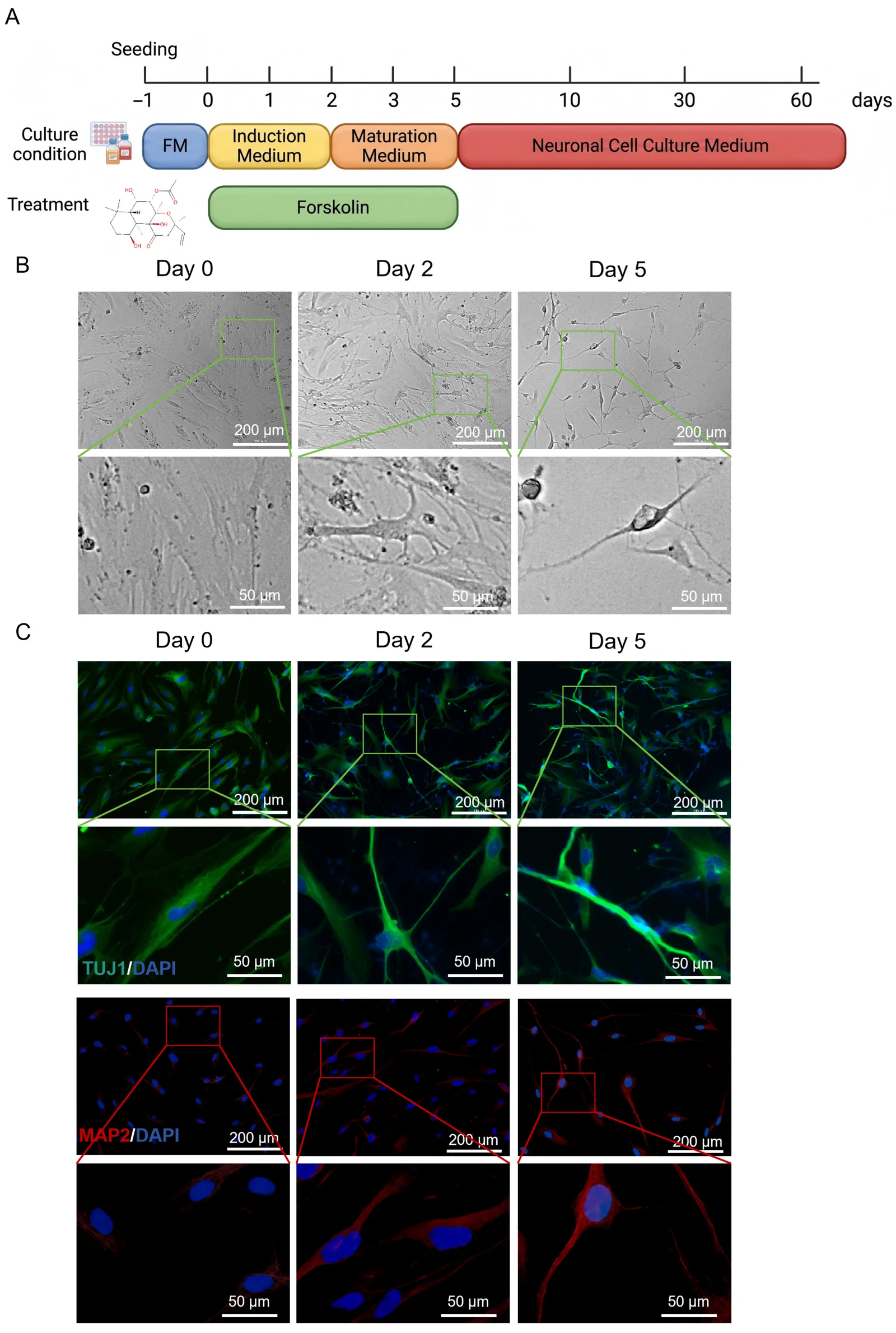
FSK-driven neuronal conversion of human somatic cells. (**A**). Schematic overview illustrating the experimental workflow for fibroblast-to-neuron conversion under the established FSK-based protocol and subsequent neuronal culture. (**B**). Representative brightfield images showing morphological changes in cells on days 0, 2, and 5 post-induction, with corresponding magnified views of selected regions. Scale bars: 200 μm; magnified views, 50 μm. (**C**). Immunofluorescence analysis of the neuronal markers TUJ1 (green) and MAP2 (red) on days 0, 2, and 5 following induction, indicating progressive acquisition of neuronal identity. Scale bars, 200 μm; magnified views, 50 μm.

Treatment of BJ human dermal fibroblasts with 10 μM FSK triggered neuronal morphological reprogramming: a bipolar, neuron-like morphology emerged within 24–36 h post-induction and became markedly enhanced by days 2–3 (Figure 1A,B). The proportion of TUJ1-positive cells increased from approximately 50% on day 2 to 90% on day 5, while the proportion of MAP2-positive cells increased from approximately 30% on day 2 to 85% on day 5 (Figure 1C and Figure S1A,B). These observations demonstrate progressive acquisition of neuronal-like morphology and neuronal marker expression during the early induction period and provide the experimental basis for subsequent proteomic and metabolomic analyses during the early induction phase.

### 3.2. Global Proteome Dynamics During FSK-Driven Transdifferentiation

**Figure 2:**
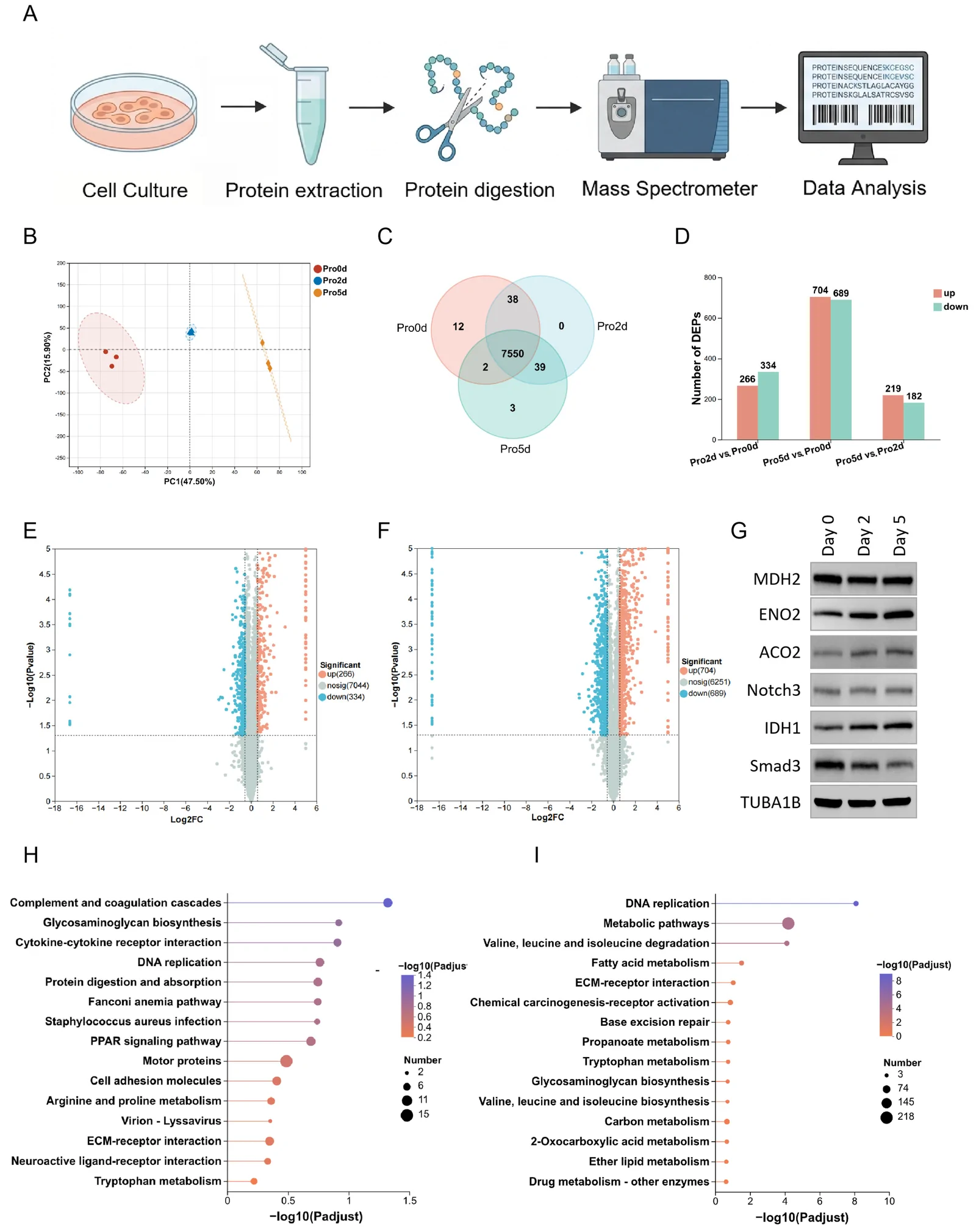
Proteomic analysis of cells at different neuronal induction stages. (**A**). Schematic of the proteomics experimental workflow. (**B**). Principal component analysis (PCA) of the proteomic data. Dashed lines indicate the PCA origin (x = 0, y = 0). Ellipses represent 95% confidence intervals for each group. PC1 and PC2 account for 47.50% and 15.90% of total variance, respectively. (**C**). Venn diagram illustrating the distribution of identified proteins across the three groups (Pro0d, Pro2d, and Pro5d). (**D**). The number of differentially expressed proteins (DEPs) in pairwise comparisons. DEPs were defined by fold change (FC) > 1.5 or < 0.67 and *p*-value < 0.05. Red and blue bars represent upregulated and downregulated proteins, respectively. (**E**,**F**). Volcano plots showing DEPs between Pro2d vs. Pro0d (**E**) and Pro5d vs. Pro0d (**F**).Vertical dashed lines are log_2_ (fold change) cut-offs (FC = 0.67 and 1.5). The horizontal dashed line marks the −log_10_(*p*-value) threshold for *p*-value = 0.05; points above the line meet *p*-value < 0.05. Red dots: significantly up-regulated genes; blue dots: significantly down-regulated genes; grey dots: non-significant genes. (**G**). Western blot analysis of key proteins at different induction timepoints (days 0, 2, and 5). TUBA1B was used as the internal reference. (**H**,**I**). KEGG pathway enrichment analysis of DEPs in the comparisons of Pro2d vs. Pro0d (**H**) and Pro5d vs. Pro0d (**I**).

Samples were collected on days 0 (Pro0d), 2 (Pro2d), and 5 (Pro5d) post-induction and subjected to data-independent acquisition (DIA)-based quantitative proteomics to establish a temporally resolved protein expression landscape during FSK-driven transdifferentiation (Figure 2A). Principal component analysis (PCA) demonstrated temporal stratification: the samples clustered tightly by timepoint with minimal intra-group dispersion, underscoring both pronounced stage-specific proteomic reorganization and excellent technical reproducibility across biological replicates (Figure 2B). Venn analysis revealed that 7550 proteins (98.8% of all proteins quantified) were stably detected across all three timepoints; only 12, 0, and 3 proteins were exclusively observed in Pro0d, Pro2d, and Pro5d, respectively (Figure 2C).

Differentially expressed protein (DEP) analysis identified 600 DEPs between Pro2d and Pro0d (266 upregulated and 334 downregulated); 1393 DEPs between Pro5d and Pro0d (704 upregulated and 689 downregulated); and 401 DEPs between Pro5d and Pro2d (219 upregulated and 182 downregulated) (fold change > 1.5 or <0.67 and *p*-value < 0.05) (Figure 2D).

Our volcano plots provide a comprehensive visualization of the distribution of DEPs across the three pairwise comparisons. The Pro2d-vs.-Pro0d comparison revealed a relatively limited number of DEPs, most of which exhibited modest fold changes (Figure 2E). In contrast, in the Pro5d-vs.-Pro0d comparison, the number of DEPs increased significantly, with more proteins exhibiting greater fold changes and higher statistical significance (Figure 2F).

To rigorously validate the accuracy of DIA quantification, we conducted Western blot analysis on six functionally coherent proteins: MDH2, ENO2, ACO2, Notch3, IDH1, and Smad3 (Figure 2G). We confirmed that their temporal abundance trends were generally consistent between Western blot quantification and proteomic detection (Figure S2A,B).

KEGG pathway enrichment analysis uncovered temporally distinct patterns of pathway representation among the DEPs. The comparison of Pro2d vs. Pro0d revealed that DEPs were significantly enriched in motor proteins, complement and coagulation cascades, glycosaminoglycan biosynthesis, cytokine–cytokine receptor interaction, and DNA replication, among other pathways. Motor proteins exhibited the highest gene count of DEPs (Figure 2H). In contrast, when comparing Pro5d vs. Pro0d, we found that DEPs showed markedly stronger and broader enrichment in DNA replication, metabolic pathways, amino acid metabolism, lipid metabolism, and extracellular matrix. Notably, DNA replication showed the strongest statistical enrichment, while metabolic pathways harbored the greatest number of DEPs (Figure 2I).

### 3.3. Global Pathway Remodeling: Metabolic Activation Coupled with Cell-Cycle Suppression

**Figure 3:**
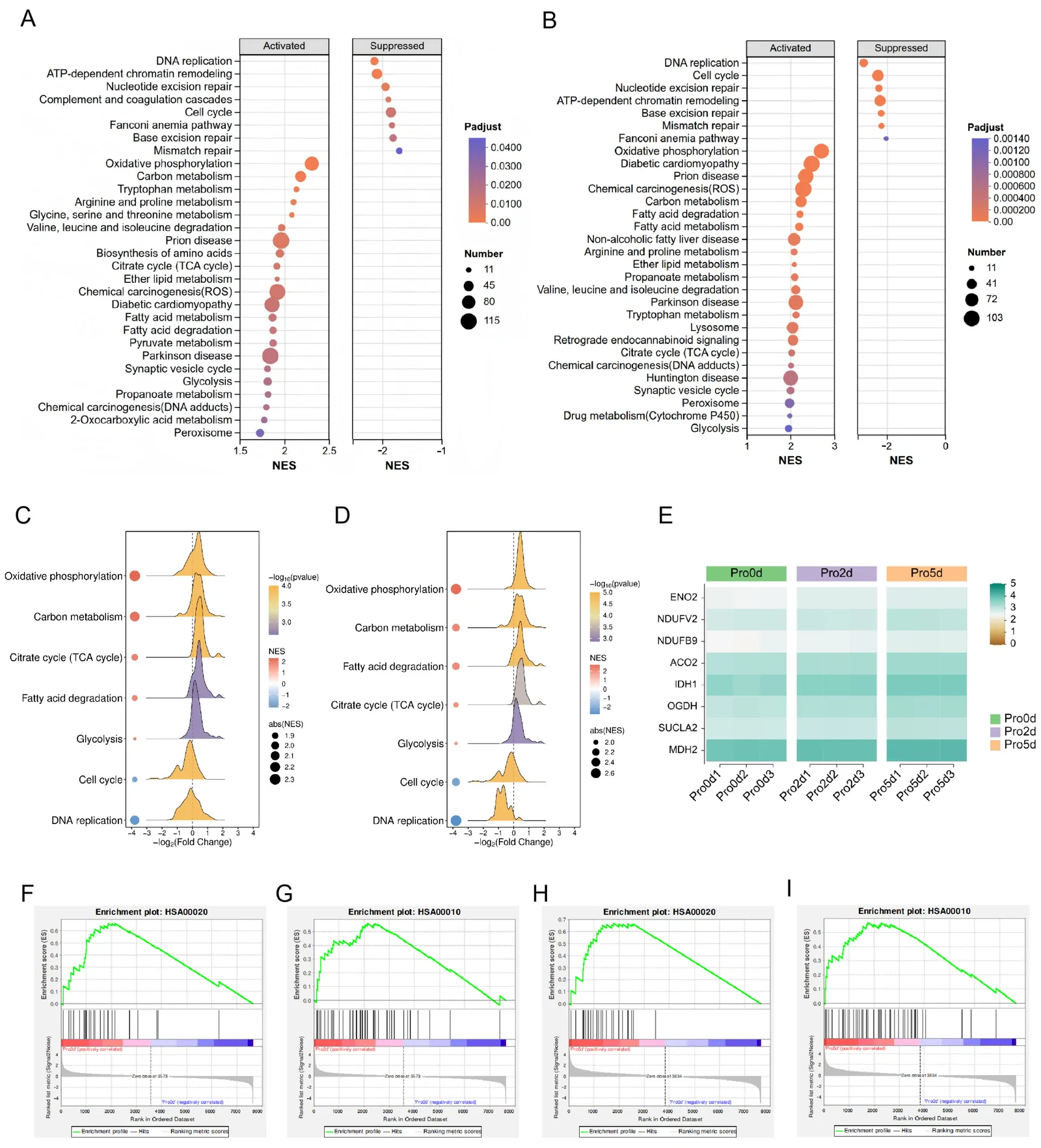
Gene Set Enrichment Analysis (GSEA) of the proteomic data. (**A**,**B**). GSEA of the global proteomic data for the Pro2d-vs.-Pro0d (**A**) and Pro5d-vs.-Pro0d (**B**) comparisons, displaying activated and suppressed pathways ranked by normalized enrichment score (NES). (**C**,**D**). Ridgeline plots of GSEA pathway distributions for the Pro2d-vs.-Pro0d (**C**) and Pro5d-vs.-Pro0d (**D**) comparisons. (**E**). Heatmap showing the expression levels of key proteins across three groups (Pro0d, Pro2d, and Pro5d). (**F**–**I**). GSEA enrichment plots for the citrate cycle (HSA00020) and glycolysis/gluconeogenesis (HSA00010) pathways in the 2 d (**F**,**G**) and 5 d (**H**,**I**) induction stages. The green line represents the enrichment profile; vertical black bars denote gene-set hits; the grey curve indicates ranking metric scores. The annotations “Pro2d (positively correlated)”, “Pro5d (positively correlated)” and “Pro0d (negatively correlated)” indicate genes at respective ends of the ranked list are positively correlated with the Pro2d and Pro5d groups, and negatively correlated with the Pro0d group.

At the early stage of transdifferentiation (Pro2d vs. Pro0d), Gene Set Enrichment Analysis (GSEA) revealed substantial positive enrichment (normalized enrichment score: NES > 0) across a wide range of energy- and biosynthesis-related pathways, including oxidative phosphorylation, carbon metabolism, the citrate acid (TCA) cycle, glycolysis, pyruvate metabolism, and fatty acid metabolism. Concurrently, multiple amino acid catabolic pathways, such as the degradation of valine, leucine, and isoleucine, as well as the metabolism of glycine, serine, and threonine, were significantly upregulated. In contrast, negatively enriched pathways (NES < 0) were predominantly associated with cell proliferation and the maintenance of genome integrity, notably including DNA replication, mitotic cell-cycle progression, and multiple DNA repair mechanisms (e.g., nucleotide excision repair, base excision repair, and mismatch repair), along with ATP-dependent chromatin remodeling (Figure 3A).

By the late stage of transdifferentiation (Pro5d vs. Pro0d), this dichotomous regulatory pattern was not only maintained but intensified. Core metabolic pathways retained strong positive enrichment, while additional functionally relevant pathways, including lysosomal biogenesis/function, endocannabinoid signaling, and cytochrome-P450-mediated metabolism, exhibited further positive enrichment. Conversely, pathways associated with the cell cycle and DNA replication/repair remained consistently suppressed (Figure 3B). Our ridge plots quantitatively illustrate the progressive shift in protein-level representation across these pathways, confirming there was time-dependent amplification of metabolic pathway enrichment and sustained attenuation of proliferative signatures during FSK-driven transdifferentiation (Figure 3C,D).

To connect pathway-level inferences with molecular effectors, we examined the expression dynamics of key enzymatic proteins across core metabolic modules. Heatmap analysis confirmed coordinated up-regulation of glycolytic (ENO2), TCA cycle (ACO2, IDH1, OGDH, SUCLA2, and MDH2), and oxidative phosphorylation (NDUFV2 and NDUFB9) components following FSK treatment (Figure 3E). Critically, this induction was both temporally progressive and highly reproducible across biological replicates, thereby providing protein-level support for the GSEA-derived metabolic enrichment pattern. Given the centrality of metabolic reprogramming in transdifferentiation, we performed temporal quantification of GSEA enrichment for two cornerstone pathways: the TCA cycle and glycolysis/gluconeogenesis. Both pathways exhibited statistically significant and monotonic increases in the normalized enrichment score over time. Specifically, on day 2, the NES values were 1.91 for the TCA cycle and 1.81 for glycolysis (Figure 3F,G). By day 5, these values had increased to 2.03 and 1.96, respectively (Figure 3H,I). This consistent, time-dependent enrichment demonstrates progressive remodeling of central carbon metabolism during the early phase of lineage conversion.

### 3.4. Temporal Evolution of the Global Metabolic Landscape

**Figure 4:**
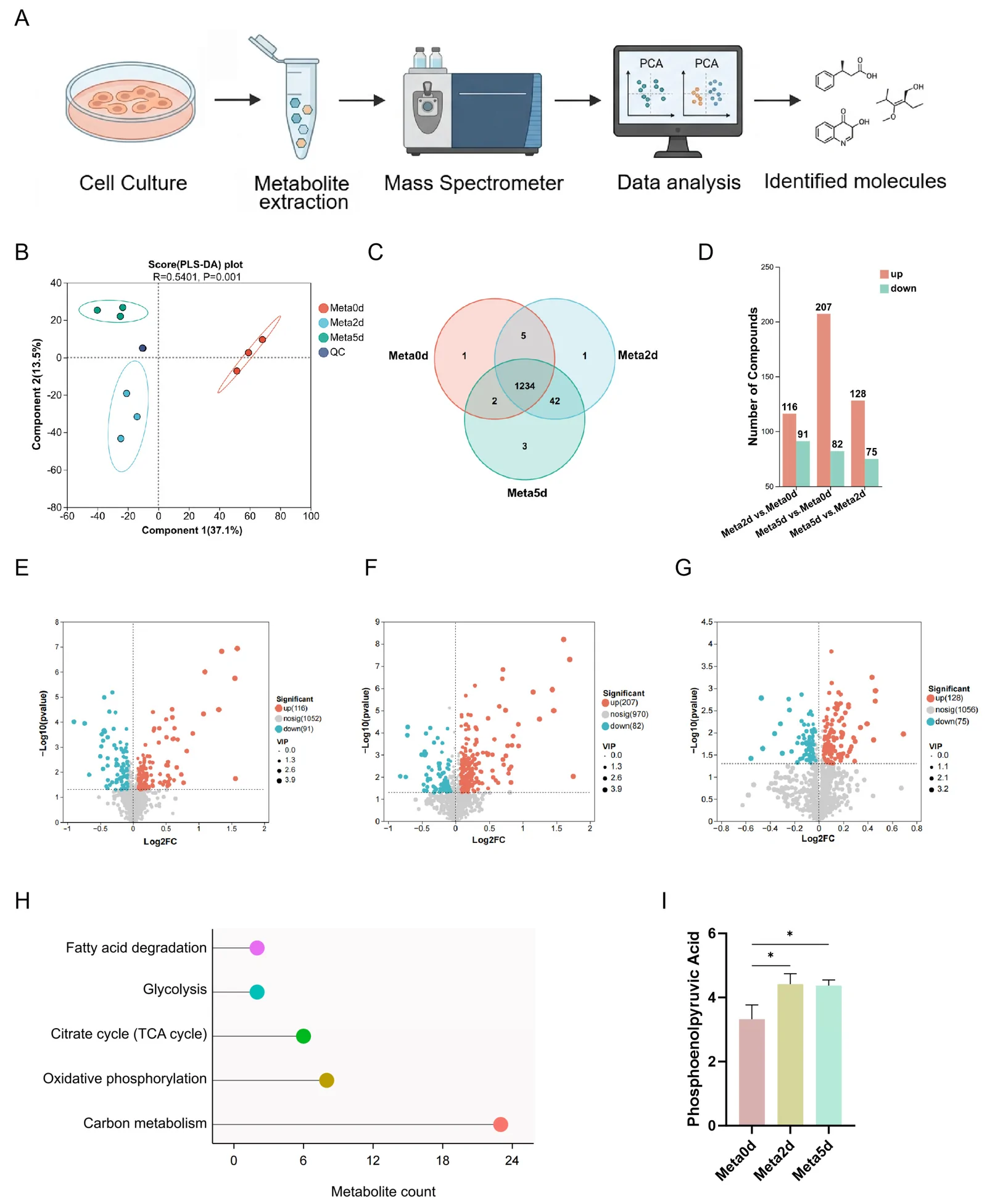
Metabolomic analysis of cells at different neuronal induction stages. (**A**). Schematic of the proteomics experimental workflow. (**B**). Partial least squares-discriminant analysis (PLS–DA) score plot of the metabolomic data. (**C**). Venn diagram illustrating the distribution of identified metabolites across the three metabolomic groups (Meta0d, Meta2d, and Meta5d). (**D**). Number of differentially expressed metabolites (DEMs) in pairwise comparisons (Meta2d vs. Meta0d, Meta5d vs. Meta0d, and Meta5d vs. Meta2d). DEMs were defined by VIP > 1 and *p*-value < 0.05. (**E**–**G**). Volcano plots of DEMs for the Meta2d-vs.-Meta0d (**E**), Meta5d-vs.-Meta0d (**F**), and Meta5d-vs.-Meta2d (**G**) comparisons. (**H**). Distribution of identified metabolites in key energy-metabolism-related pathways. (**I**). Abundance of the key differential metabolite phosphoenolpyruvic acid at different induction stages (Meta0d, Meta2d, and Meta5d). Data are presented as means ± SD (*n* = 3 biological replicates), * *p*-value < 0.05.

To systematically characterize the metabolic reprogramming dynamics associated with fibroblast-to-neuron conversion under the established FSK-based conversion protocol, we performed untargeted metabolomic profiling on cellular extracts harvested on days 0 (Meta0d), 2 (Meta2d), and 5 (Meta5d) (Figure 4A).

The PLS-DA score plot indicates high analytical robustness: the biological replicates and quality control (QC) samples exhibit tight intra-group clustering, while the three timepoints, Meta0d, Meta2d, and Meta5d, form a statistically significant, unidirectional gradient along principal component 1 (PC1, 37.1% variance explained). ANOSIM further confirms significant differences among groups (R = 0.5401, *p* = 0.001), where the R value indicates that inter-group variation exceeds intra-group variation (Figure 4B). Permutation testing of the PLS-DA model further confirmed the absence of overfitting (Figure S3). This ordered separation indicates that transdifferentiation proceeds through a reproducible sequence of metabolic states. Moreover, the Venn diagram shows that 1234 metabolites (86.4% of the 1428 metabolites that were confidently identified and quantified) were consistently detected under all conditions. This result suggests that the metabolic differences across different timepoints mainly arise from the changes in the abundance of shared metabolites (Figure 4C). OPLS-DA models were constructed for each comparison group, and then permutation tests were conducted to assess model validity; the results verified that the established models were robust and reliable (Figure S4A–F).The criteria for defining differentially expressed metabolites (DEMs) were VIP > 1 (from OPLS-DA) and *p*-value < 0.05. The resulting DEM profiles reveal a progressive and non-redundant escalation: in Meta2d vs. Meta0d, 207 DEMs were identified (116 upregulated, 91 downregulated). In Meta5d vs. Meta0d, 289 DEMs (207 upregulated, 82 downregulated) were obtained, showing an ample effect size and higher statistical confidence at the endpoint. For Meta5d vs. Meta2d, there were 203 DEMs (128 upregulated, 75 downregulated) (Figure 4D). Together, these data demonstrate that metabolic remodeling remains highly dynamic and progressively intensifies throughout the mid-to-late stages of transdifferentiation.

Volcano plot analysis revealed that the DAMs between Meta2d and Meta0d predominantly clustered near the significance and fold-change thresholds. In the Meta5d vs. Meta0d group, the number of DAMs increased, and more metabolites exhibited greater fold changes and higher statistical significance. Finally, substantial metabolic divergence persisted between Meta5d and Meta2d, with numerous metabolites remaining significantly differentially abundant (Figure 4E–G).

To relate the differential metabolites to central energy metabolism pathways, we mapped the quantified metabolites onto KEGG-annotated core energy pathways (Figure 4H). Carbon metabolism showed the highest metabolite coverage (24 metabolites), followed by oxidative phosphorylation (6), TCA cycle (4), glycolysis (3), and fatty acid degradation (2). Crucially, phosphoenolpyruvate (PEP), a node metabolite that controls the glycolytic flux into biosynthetic branches and regulates pyruvate kinase activity, exhibited a monotonic, statistically significant accumulation. The level of PEP in Meta2d increased significantly and remained at a relatively high level at Meta5d relative to Meta0d (Figure 4I).

### 3.5. Integrated Protein–Metabolite Network Reveals Coordinated Upregulation Across Glycolysis and the TCA Cycle

**Figure 5:**
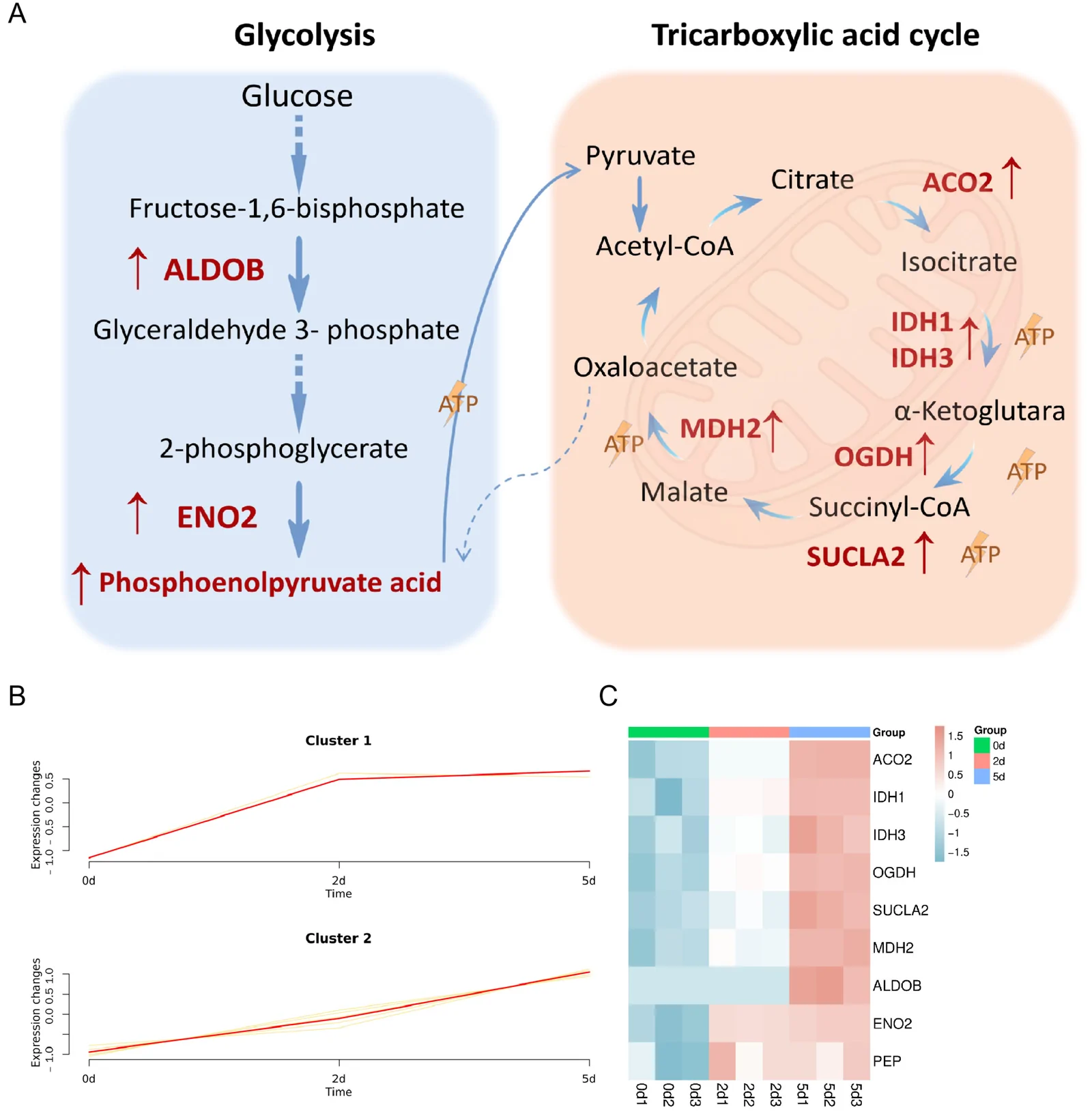
Integrated proteomic and metabolomic analysis of central carbon metabolism during neuronal induction. (**A**). Integrated proteomics and metabolomics network analysis of glycolysis and the TCA cycle. Proteins and metabolites highlighted in red with upward arrows are significantly upregulated. In the glycolysis pathway, blue solid arrows represent direct biochemical reactions, and blue dashed arrows represent indirect effects. Blue arrows in the tricarboxylic acid cycle mark cyclic-reaction flow. (**B**). Clustered temporal expression patterns of key glycolysis and TCA cycle DEPs and DEMs. Yellow lines represent expression trends of individual DEPs/DEMs; the red line shows the overall expression trend within each cluster. (**C**). Heatmap showing the expression patterns of key DEPs and DEMs involved in glycolysis and the TCA cycle across different induction stages.

To integrate the protein and metabolite level changes across central carbon metabolism, we constructed a biochemically constrained, directional protein–metabolite network mapping the glycolysis–TCA cycle interface (Figure 5A). In the process of glycolysis, both aldolase B (ALDOB) and enolase 2 (ENO2) were significantly upregulated at the protein level, concomitant with a marked increase in PEP. In mitochondrial metabolism, key enzymes of the TCA cycle—including aconitase 2 (ACO2), isocitrate dehydrogenases (IDH1 and IDH3 subunits), oxoglutarate dehydrogenase (OGDH), succinate-CoA ligase ADP-forming subunit beta (SUCLA2), and malate dehydrogenase 2 (MDH2)—exhibited consistent upregulation at the protein level. These findings demonstrate that molecules associated with glycolysis and the TCA cycle display coordinated regulation across both the proteomic and metabolomic levels during FSK-driven transdifferentiation.

To further characterize the temporal expression dynamics of key regulatory molecules, we applied trend-based clustering to the core proteins and PEP identified through integrated proteomic and metabolomic analysis (Figure 5B). This analysis revealed two distinct, monotonically increasing expression clusters: One exhibited rapid upregulation between Meta0d and Meta2d, followed by a sustained elevation until Meta5d. The other displayed a gradual but continuous accumulation from Meta0d to Meta5d.

To further validate the coordinated upregulation of glycolytic and mitochondrial TCA cycle components during FSK-driven neuronal transdifferentiation, we generated a time-resolved integrative heatmap encompassing both protein abundance and metabolite levels. The results showed that, compared to the day 0 control group, key glycolytic proteins (ENO2 and ALDOB), TCA cycle-related enzymes (ACO2, IDH1, OGDH, SUCLA2, and MDH2), and the metabolite PEP all exhibited progressive, time-dependent upregulation, showing elevated expression on day 2, with significant increases observed by day 5 (Figure 5C). Altogether, these results suggest that metabolic reprogramming during the fibroblast-to-neuron conversion process is characterized by coordinated enhancement of glycolysis, mitochondrial TCA cycle activity, and oxidative phosphorylation.

## 4. Discussion

Cell transdifferentiation is a pivotal strategy in direct reprogramming, offering a promising approach for cell-based therapeutics for neurodegenerative disorders [16]. Previous studies have established that specific combinations of transcription factors or small molecules can directly reprogram human dermal fibroblasts into functional neurons. This process is associated with transcriptional silencing of fibroblast identity genes and the extensive induction of neuron-specific gene programs [17,18,19]. Our previous study demonstrated that FSK monotherapy alone is sufficient to drive rapid, direct neuronal reprogramming. Mechanistically, this conversion is regulated by the cAMP–CREB1–JNK signaling axis [15]. However, the temporal proteomic and metabolic adaptations accompanying the critical early period of cell fate conversion remain incompletely characterized. Therefore, we integrated proteomics and untargeted metabolomics to comprehensively characterize the dynamic molecular landscape during fibroblast-to-neuron conversion, revealing coordinated alterations in metabolic remodeling and proteome reorganization associated with neuronal fate transition.

A central finding of this study is the temporally coordinated suppression of the cell cycle associated programs and enrichment of energy metabolism related pathways during the earliest phase of transdifferentiation. Proteome-wide GSEA revealed a sustained negative enrichment of DNA replication, mitotic cell cycle, and DNA damage repair pathways on days 2 and 5 after induction. This pattern is consistent with progressive suppression of proliferative and cell-cycle-associated molecular programs during early transdifferentiation. Notably, this observation is consistent with our previously published single-cell transcriptomic analysis of the same neuronal conversion system, in which an early cell population expressed cell-cycle-associated genes, including MKI67, CDK1, and CENPF, whereas cells entering a subsequent intermediate neuronal-lineage state showed downregulation of cell-cycle-related genes accompanied by increased expression of neuronal differentiation-associated genes [15]. Previous studies in human fibroblasts also support an antiproliferative effect of forskolin/cAMP signaling. Meng et al. reported that forskolin at 1–10 µM dose-dependently inhibited DNA synthesis and proliferation in human dermal fibroblasts, whereas Perez-Aso et al. similarly observed reduced proliferation of primary normal human dermal fibroblasts following forskolin-mediated elevation of intracellular cAMP [20,21]. Together, these findings support the notion that suppression of proliferative programs accompanies the transition toward a differentiated cell state. Consistent with previous reports, attenuation of cell-cycle-associated activity has been frequently observed during lineage commitment, whereas sustained proliferative signaling may interfere with cellular differentiation processes [22].

In marked contrast, energy metabolism pathways, including oxidative phosphorylation, glycolysis, the TCA cycle, and carbon metabolism, showed the most significantly and temporally coherent positive enrichment among all regulated pathways. GSEA, mountain plots, and quantitative heatmaps convergently support this conclusion. Notably, several key enzymes involved in glycolysis and the TCA cycle, including ENO2, ACO2, IDH1, IDH3, OGDH, SUCLA2, and MDH2, showed increased protein abundance, further supporting coordinated remodeling of central carbon metabolism. Collectively, these findings support a model in which metabolic reprogramming represents a coordinated and biologically relevant component of early cell-state transition rather than a passive metabolic adaptation [23,24]. These metabolic alterations may contribute to the establishment of a permissive cellular environment for neuronal conversion, although their direct regulatory roles require further functional validation.

Metabolomics analysis provides orthogonal validation of the prior findings. PLS-DA uncovered a temporally resolved, progressive reorganization of the global metabolic landscape, with a substantial expansion in the number of significantly dysregulated metabolites by day 5. Strikingly, PEP levels increased rapidly upon induction and were stably maintained at elevated concentrations thereafter. Although changes in the abundance of a single metabolite cannot be equated with altered enzymatic flux, the combined up-regulation of glycolytic enzymes and key enzymes of the TCA cycle, as well as the continuous positive enrichment of related pathways in GSEA, collectively supports the existence of a systematic reconfiguration of central carbon metabolism during transdifferentiation.

Integrated proteomic and metabolomic analysis revealed functionally coherent regulation across central metabolic pathways. Within the integrated molecular network, proteins associated with glycolysis, including ALDOB and ENO2, showed synchronous upregulation with PEP, while core TCA cycle enzymes (ACO2, IDH1, IDH3, OGDH, SUCLA2, and MDH2) exhibited concordant induction at the protein level. Together, these coordinated changes support directional, biphasic remodeling of central carbon metabolism, which involves an initial enhancement of glycolytic flux coupled with a subsequent amplification of mitochondrial oxidative capacity. Moreover, temporal trend clustering revealed two distinct kinetic profiles: early-responding molecules that surged rapidly and plateaued at elevated levels and late-responding molecules exhibiting a progressive, monotonic accumulation over time. This temporally stratified activation pattern implies there are stage-specific functional roles for metabolic reprogramming during transdifferentiation. Furthermore, our proteomic and metabolomic data revealed highly overlapping molecular profiles during the early stage of transdifferentiation, suggesting that the initial cell state transition is not driven by extensive synthesis of proteins or metabolites but rather by the dynamic abundance remodeling of pre-existing molecular networks to regulate cell fate.

While this study provides a comprehensive characterization of early transdifferentiation through integrated proteomic and metabolomic analyses, several important limitations demand careful consideration. Firstly, the temporal coverage is restricted to days 0–5, thereby excluding critical later-stage processes, such as morphological maturation. Secondly, although integrative analysis identified key hub enzymes (e.g., ALDOB, ENO2, ACO2, and IDH1) whose coordinated regulation is strongly correlated with metabolic reprogramming, their causal contributions to transdifferentiation remain unproven. Definitive validation will require orthogonal functional interrogation in relevant cellular models. Thirdly, although the present study provides a comprehensive temporal multi-omics landscape during FSK-driven fibroblast-to-neuron conversion, the potential contributions of individual components within the induction medium, including serum replacement, KOSR, B27, and other supplements, cannot be completely excluded. Future studies employing parallel conversion systems with matched medium conditions in the presence or absence of FSK will be necessary to further distinguish the specific molecular effects of FSK signaling from those mediated by environmental cues.

In summary, this study establishes the key characteristics of FSK-driven fibroblast-to-neuron transdifferentiation during its early phase. This process is marked by a concerted suppression of the cell-cycle machinery. Meanwhile, energy metabolism pathways, including glycolysis, the TCA cycle, and oxidative phosphorylation, are coordinately upregulated. An integrated proteomic and metabolomic analysis identified sustained co-activation of the glycolysis–mitochondrial oxidative axis as a core molecular signature underlying this reprogramming event. Collectively, these findings provide a systems-level mechanistic framework for small-molecule-driven cell-fate conversion and establish metabolic reprogramming as a functionally integral and therapeutically targetable axis for enhancing transdifferentiation efficiency and neuronal lineage fidelity.

## Data Availability

The mass spectrometry proteomics data have been deposited to the ProteomeXchange Consortium (https://proteomecentral.proteomexchange.org, accessed on 8 July 2026) via the iProX partner repository with the dataset identifier PXD080722. The metabolomics data have been deposited to MetaboLights (https://www.ebi.ac.uk/metabolights, accessed on 6 July 2026) repository with the study identifier MTBLS15000.

## Supplementary Materials

The following supporting information can be downloaded at https://www.mdpi.com/article/10.3390/cimb48090871/s1.
