# Supplementary material for: Dynamic Proteomic and Metabolomic Analysis Reveals Metabolic Reprogramming During Early Neuronal Transdifferentiation of Human Fibroblasts Driven by Forskolin
Source: Curr Issues Mol Biol. 2026 Aug 27;48(9):871. doi: 10.3390/cimb48090871 (PMC13605807; doi:10.3390/cimb48090871)
Supplement: Supplementary file 1 [file cimb-48-00871-s001.zip › cimb-4488464-supplementary.pdf]

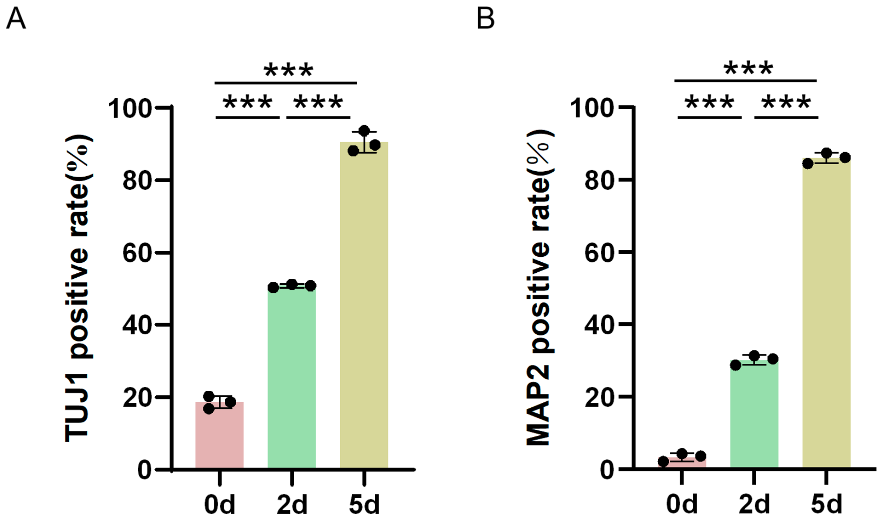

Figure S1. Quantification of TUJ1-positive (**A**) and MAP2-positive (**B**) cell proportions at 2 and 5 days after induction. Data are presented as means ± SD (n = 3 biological replicates). Statistical significance was determined using one-way ANOVA followed by Tukey's multiple comparisons test. *$p < 0.05$, **$p < 0.01$, ***$p < 0.001$.

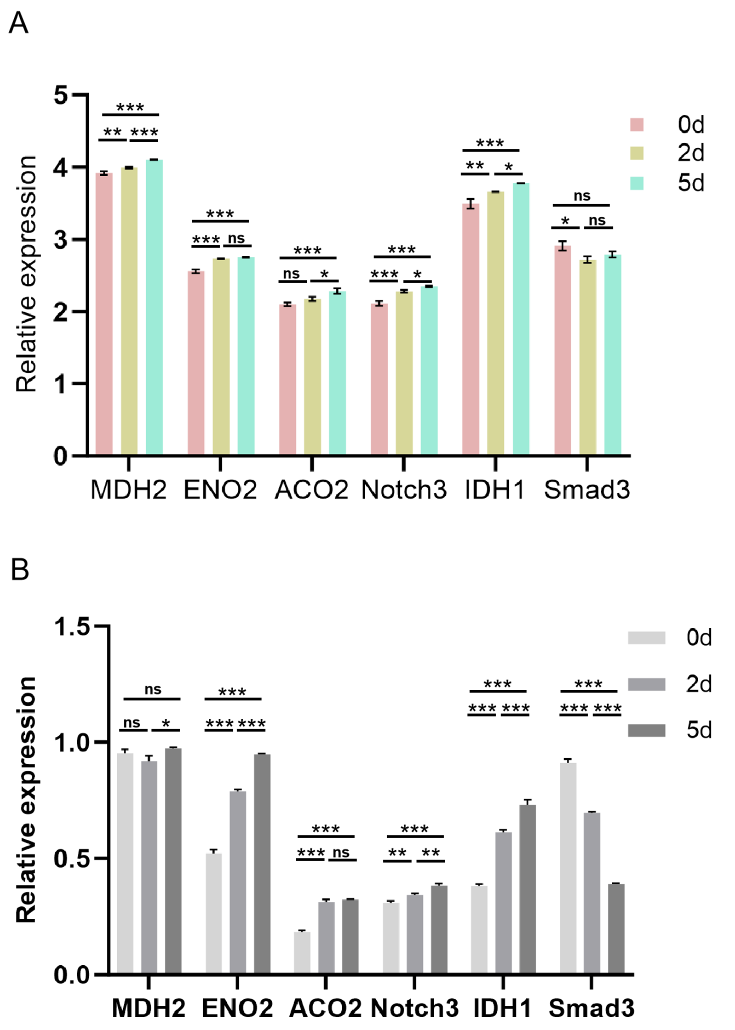

Figure S2. Temporal expression analysis of selected proteins during differentiation. (**A**) Proteomic analysis of MDH2, ENO2, ACO2, Notch3, IDH1, and Smad3 at days 0, 2, and 5. (**B**) Western blot quantification of the corresponding proteins, normalized to the loading control. Data are mean ± SD (n = 3). Statistical significance was determined using one-way ANOVA followed by Tukey's multiple comparisons test. *$p < 0.05$, **$p < 0.01$, ***$p < 0.001$; ns, not significant.

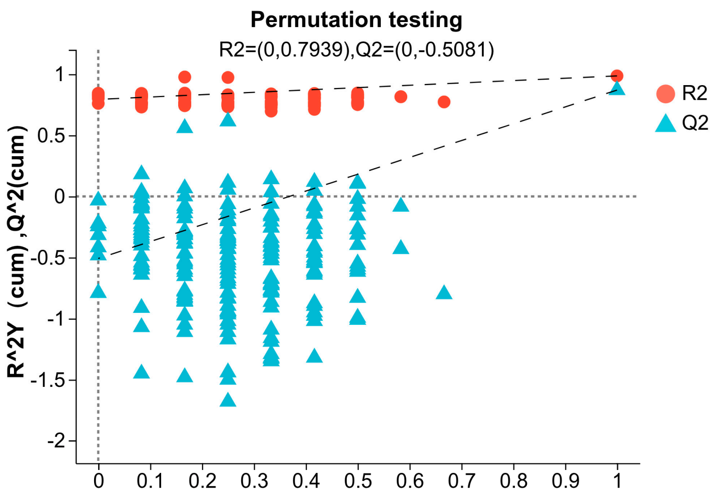

Figure S3. Permutation test of the PLS-DA model based on all metabolomics samples. Permutation testing was performed to validate the PLS-DA model. Red circles represent R^2 values, and cyan triangles represent Q^2 values.

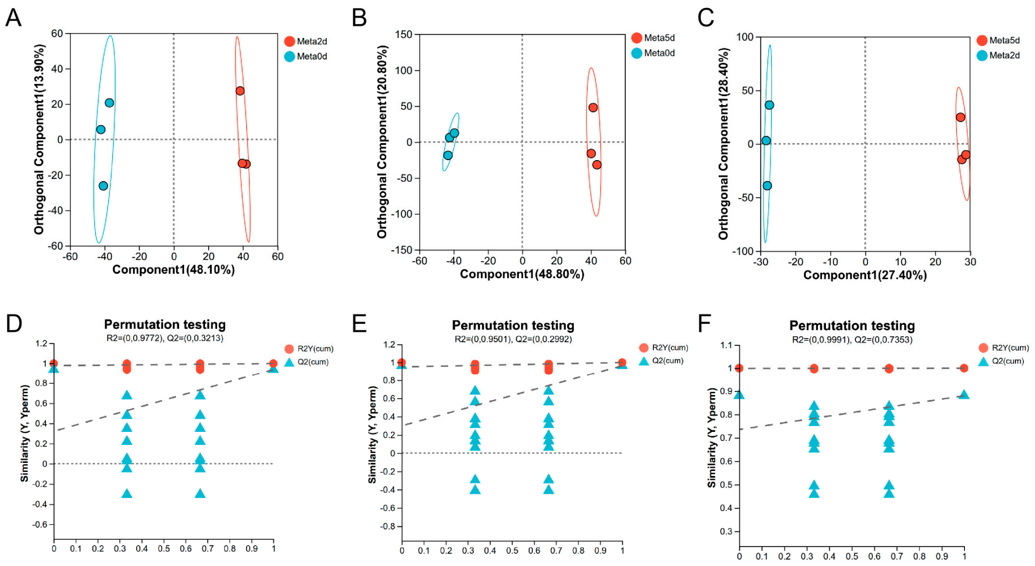

Figure S4. OPLS-DA analysis and permutation validation of metabolic profiles. (**A–C**) OPLS-DA score plots for Meta2d vs. Meta0d (**A**), Meta5d vs. Meta0d (**B**), and Meta5d vs. Meta2d (**C**). (**D–F**) Permutation tests for the corresponding OPLS-DA models. Red points indicate original R^2Y and blue triangles represent represent Q^2 values. Negative Q^2 intercepts indicate models are not overfitted.
